# Single-Cell Transcriptome Analysis Identifies Subclusters with Inflammatory Fibroblast Responses in Localized Scleroderma

**DOI:** 10.3390/ijms24129796

**Published:** 2023-06-06

**Authors:** Giffin Werner, Anwesha Sanyal, Emily Mirizio, Theresa Hutchins, Tracy Tabib, Robert Lafyatis, Heidi Jacobe, Kathryn S. Torok

**Affiliations:** 1 Department of Pediatrics (Rheumatology), University of Pittsburgh, Pittsburgh, PA 15224, USA; wernergj@upmc.edu (G.W.); sanyala@upmc.edu (A.S.); emmirizio@gmail.com (E.M.); hutchinstr@upmc.edu (T.H.); 2 Division of Rheumatology and Clinical Immunology, University of Pittsburgh, Pittsburgh, PA 15261, USA; trt28@pitt.edu (T.T.); lafyatisra@upmc.edu (R.L.); 3 Department of Dermatology, University of Texas Southwestern, Dallas, TX 75390, USA

**Keywords:** localized scleroderma, single-cell RNA sequencing, fibroblasts, morphea, CXCR3 ligands, CXCL9, CXCL10, IL-6, skin, cell communication, fibrosis, inflammation

## Abstract

Localized scleroderma (LS) is an autoimmune disease with both inflammatory and fibrotic components causing an abnormal deposition of collagen in the skin and underlying tissue, often leading to disfigurement and disability. Much of its pathophysiology is extrapolated from systemic sclerosis (SSc) since the histopathology findings in the skin are nearly identical. However, LS is critically understudied. Single-cell RNA sequencing (scRNA seq) technology provides a novel way to obtain detailed information at the individual cellular level, overcoming this barrier. Here, we analyzed the affected skin of 14 patients with LS (pediatric and adult) and 14 healthy controls. Fibroblast populations were the focus, since they are the main drivers of fibrosis in SSc. We identified 12 fibroblast subclusters in LS, which overall had an inflammatory gene expression (IFN and HLA-associated genes). A myofibroblast-like cluster (SFRP4/PRSS23) was more prevalent in LS subjects and shared many upregulated genes expressed in SSc-associated myofibroblasts, though it also had strong expression of CXCL9/10/11, known CXCR3 ligands. A CXCL2/IRF1 cluster identified was unique to LS, with a robust inflammatory gene signature, including IL-6, and according to cell communication analysis are influenced by macrophages. In summary, potential disease-propagating fibroblasts and associated gene signatures were identified in LS skin via scRNA seq.

## 1. Introduction

Localized scleroderma (LS) or morphea is a rare autoimmune disease affecting the skin and underlying connective tissue in adults and children, with an annual incidence of 0.4 to 2.7 cases per 100,000 population [1,2]. LS is thought to be an inflammatory driven fibrotic disease having a similar endpoint to that of systemic sclerosis, with dense collagen deposition in the dermis and underlying connective tissue. In LS, this results in linear bands of fibrosis deep in the connective tissue, and in growing children, leads to joint contractures and disability through their life spectrum, among other cosmetic and noncosmetic issues. The excessive extracellular matrix deposition in the skin is thought to be a result of inflammation-triggered fibroproliferation and differentiation of fibroblasts into myofibroblasts [3,4,5], following suit of systemic sclerosis (SSc) pathophysiology [6,7,8,9,10]; however, this is only extrapolated to LS and remains unknown.

In general it is conceptualized that after injury or tissue damage, fibroblasts in the connective tissue become activated and differentiate into activated fibroblasts or myofibroblasts, the latter expressing alpha smooth muscle actin (α-SMA, *ACTA2*) with the contractile capacity of smooth muscle cells and collagen synthesis ability of fibroblasts, and create extracellular matrix remodeling to enhance wound healing [11,12,13]. Myofibroblasts typically regress from the site of injury in normal healing processes [14]; however, in scleroderma and other fibrotic conditions, such as idiopathic pulmonary fibrosis, they tend to persist and play a role in abnormal ECM composition and fibrosis [7,9,15,16,17,18,19,20,21]. Myofibroblasts and other transitioning fibroblasts subsets have been of recent interest in systemic sclerosis (SSc) pathogenesis, specifically with the advent of single-cell RNA sequencing technology identifying the heterogenous nature of fibroblasts subsets in the skin and lungs of SSc patients. For example, in SSc skin a subset of SFRP2+ fibroblasts which express SFRP4 were identified to be myofibroblasts upregulated in genes such as *ACTA2*, indicating its role in fibrosis [6,7,8,9,10,22,23,24]. We surmise that there is similar heterogeneity to the fibroblast populations within localized scleroderma, with some overlapping clusters but a few unique subsets compared with SSc. The few morphea patients (*n* = 3) who were included in a recent large scRNA-seq skin study focused on a diverse clinical spectrum of SSc patients [7] and our groups validation study of fresh vs. cryo-frozen skin (*n* = 3) scRNA-seq study in LS [25] provide feasibility of scRNA seq in LS tissue. This study was designed to advance to the next step, to comprehensively investigate the heterogeneity of fibroblasts populations within LS subjects, encompassing pediatric- and adult-onset LS and the main LS clinical subtypes (linear, generalized, plaque), using scRNA seq of the skin mediated by a pediatric/adult rheumatology–dermatology collaboration.

## 2. Results

### 2.1. Transcriptome Profiles Identify Unique Subpopulations of Fibroblast Populations with Shift in LS towards Inflammatory Phenotype

Skin biopsies from fourteen patients with LS (eight adult and six pediatric) and from fourteen age-, sex- and ethnicity-matched healthy controls (*n* = 14 LS, *n*=14 HC; *n*=28 total) were obtained to perform scRNA seq (Table 1). Patients were 57% female, mostly non-Hispanic white (78%), with average age 15 years old and 55 years old at time of biopsy for pediatric- and adult-onset LS, respectively. The common disease subtypes of LS were included: linear trunk/limb, linear face/scalp, generalized morphea and circumscribed morphea (Table 1 and Table S1). 

**Figure 1:**
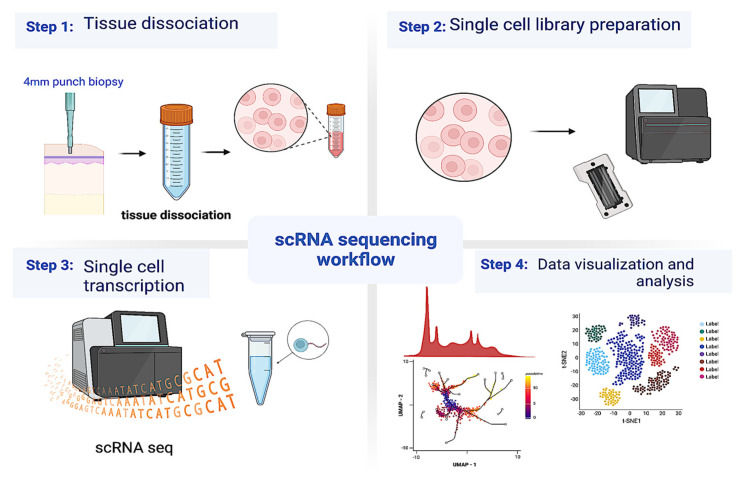
Overview of the experimental setting. Punch skin biopsy samples (4 mm) were collected from LS and healthy donors from two clinical sites (UTSW and Pitt) and subjected to tissue dissociation using the manufacturer’s protocol, and single cells were collected. The single-cell suspension was loaded on to the 10x Genomics^®^ platform following single cell library preparation for single-cell RNA sequencing (scRNA seq) and the data obtained were analyzed using Seurat on an R platform. Different visualizations were used to represent the data and predict networks and cell–cell interactions between different cell types.

Two 4 mm punch biopsies of the affected area were taken at the time of collection, one allocated for scRNA seq and the other for histological processing. scRNA seq analysis from these enzymatically digested skin biopsies were used to study the transcriptomic profile of fibroblasts. A total of 60,966 cells from fourteen LS patients (28,812 total cells—six pediatric and eight adult) and fourteen healthy (32,154 total cells—six pediatric and eight adult) age-matched controls were obtained from our scRNAseq workflow (Figure 1). 

These total cells analyzed with Seurat provided unsupervised clustering of 43 cell populations (Figure S1A), all of which included both healthy and LS disbursement (Figure S1B), and then were categorized into 14 main groups of cells using annotations from published gene expression profiles for the different cell types (Figure S1C) (Table S2). Our primary cells of interest, the fibroblasts, were characterized by COL1A1, COL1A2, and PDGFRA expression identified within these clusters (Figure S1D). 

**Figure 2:**
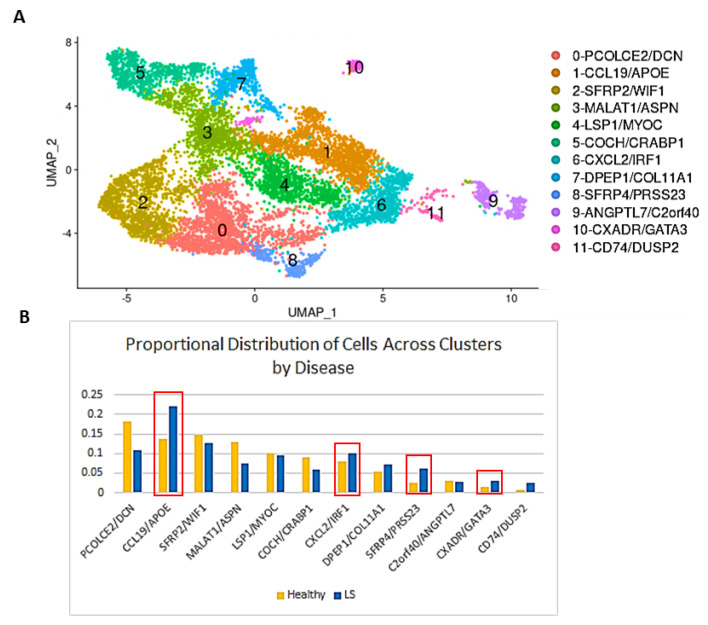
Transcriptome profiles identify unique subpopulations of fibroblast populations with a shift in LS towards a specific phenotype. (**A**) UMAP plotting 9192 fibroblast cells clustered into 12 unique subclusters among all fibroblasts within LS and healthy. (**B**) Bar graph showing the distribution of LS cells across the subclusters compared with that of healthy cells. Clusters 1, 8, and 10 had a notably higher distribution of LS cells compared with healthy (noted by red box outline).

**Figure 3:**
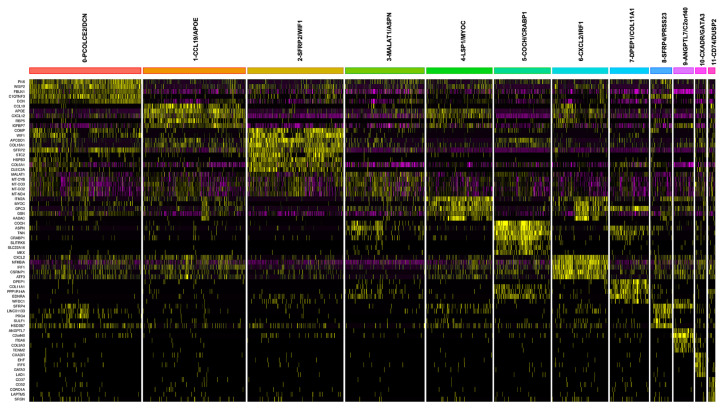
Heatmap of top fibroblast cluster-defining differentially expressed genes (DEGs). Heatmap of top 5 genes of the cluster-defining DEGs among all fibroblasts containing both LS and healthy cell populations, with cluster-defining DEGs used for nomenclature of the 12 subclusters. Yellow on the heatmap shows most highly upregulated and pink shows the most downregulated genes.

Other cells were also clustered using known markers, as follows: keratinocytes were identified by cell cluster expression of KRT1 [26], KRT14 [27], and KRT5 [28]; pericytes [29] by RGS5, CSPG4, and PDGFRB; T cells by CD3D [30], CD3E, CD8A, and CD4 [31]; macrophages by CD163 [32] and AIF1 [33]; dendritic cells (DCs) [34] by CD1C and FCER1A; natural killer (NK) cells by NKG7 [35]; melanocytes by PMEL [36]; B cells by IGJ and MS4A1 [37]; mast cells by TPSAB1 [38]; eccrine cells by AQP5 [39] and DCD [30]; and endothelial and lymphatic endothelial cells by ACKR1 [30] and LYVE1, respectively [40]. Further comparison of the 9192 total skin fibroblasts comprised of patient- and control-derived samples identified 12 distinct subclusters, which were annotated using the top DEGs and references to literature (Figure 2, Figure 3 and Figure S2; Table S3). 

Cluster 0 (PCOLCE2/DCN) highly expressed common fibroblast markers and comprised the largest group of fibroblasts in both healthy and LS patients [9]. Cluster 1 was highly expressive of CCL19 and APOE [41,42], markers for proinflammatory fibroblasts thought to play a role in the activation of T cells and other immune cells in the periphery of the lesions to aid inflammation and fibrosis [8]. A CCL19/APOE cluster has previously been reported to be upregulated in SSc fibroblasts [8]. Cluster 2 highly expressed SFRP2 and WIF1, both known to be major fibroblast subset markers [9]. Cluster 3 was a low-quality cluster expressing MALAT1 and ASPN. Cluster 4 expressed MYOC and LSP1, a marker of another known fibroblast subpopulation [24]. Cluster 5 consists of classic markers for dermal papillae (COCH/CRABP1) [8,41], and Cluster 6 was highly expressing CXCL2/IRF1, a potent proinflammatory cluster that could help in recruitment of neutrophils and macrophages to the site of the lesions [43]. Clusters 7–10 mostly consisted of smaller clusters but displayed distinct expression and subtypes, particularly of transitioning markers. Cluster 7 (DPEP1/COL11A1) was identified as dermal sheath cells, and Cluster 8 (SFRP4/PRSS23) showed some of the previously identified markers of myofibroblast cells (PRSS23, SFRP4, MFAP5) [8]. Cluster 9 (ANGPTL7/C2orf40) [9] defined a well-segregated and previously described but uncharacterized subset of fibroblasts [8], which may be intermediary between dermal papillae and dermal sheaths. Cluster 10 (CXADR/GATA3) represents a possible proliferative cell type, and Cluster 11 was a small cluster expressing DUSP2 and CD74 [44,45]. 

Comparison of the LS cell distribution across the subclusters with the healthy-control cell distribution found a higher frequency in three particular clusters. Specifically, Cluster 1 (immune response, CCL19/APOE), Cluster 8 (transitioning/wound healing/contractile, SFRP4/PRSS23), and Cluster 10 (proliferation regulation, CXADR/GATA3) were noted to be higher in distribution amongst LS cells compared with healthy cells (Figure 2B; Table S4). 

### 2.2. LS Fibroblasts Show Upregulation of Genes Involved in Immune Response, Especially in the Interferon and CXCL Pathways

**Figure 4:**
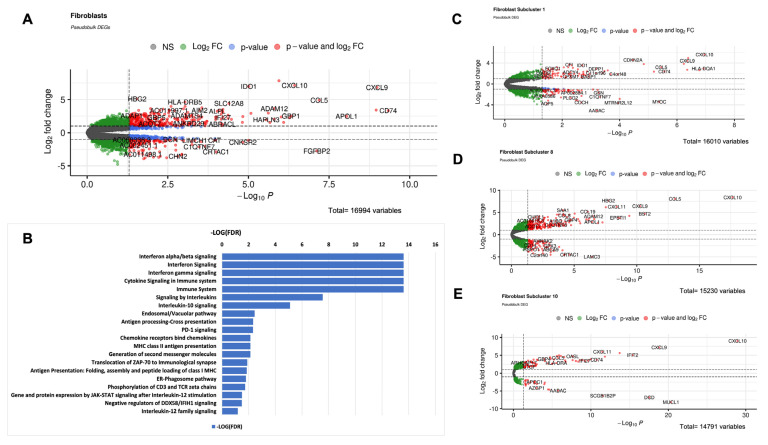
LS fibroblasts show upregulation of genes involved in immune response, especially in the interferon pathways. (**A**) Volcano plot of differentially expressed genes between LS and healthy across all fibroblasts, using Libra’s pseudobulk EdgeR method. (**B**) Bar graph of Reactome pathway −log(FDR) found from LS upregulated genes which showed high upregulated expression of inflammatory genes, including interferon gamma and cytokine signaling pathways. Volcano plots of differentially expressed genes between LS and healthy across (**C**) Cluster 1 fibroblasts, (**D**) Cluster 8 fibroblasts, and (**E**) Cluster 10 fibroblasts, using Libra’s pseudobulk EdgeR method.

Differential gene expression (DEG) analyses between the LS and the healthy controls in total fibroblast cells and within subclusters, with a focus on Clusters 1, 8, and 10 since these were more frequently populated by LS subjects, were examined. Findings include upregulated genes related to CXCR3 ligands (CXCL9, CXCL10, CXCL11), INF-pathway (IRF1, IFIT2, IL6, CCL5, CCL2), and HLA expression (HLA-B, HLA-C HLA-DRB1, HLA-DRA) in LS fibroblasts (total and subclusters 1, 8, and 10), compared with the healthy controls (Figure 4A,C–E). Cluster 8 (SFRP4/PRSS23), our identified myofibroblast-like population, also highly expressed ADAM12 (Figure 4D), a marker highly expressed in SFRP2/SFRP4 myofibroblasts in SSc [9].

Biological interaction pathway analysis performed with Reactome to predict which pathways are differentially regulated in LS also supported these observations with IFNαβ, IFNγ, Interleukin signaling, Antigen processing-cross presentation, and MCH class II antigen presentation processes all enhancing in LS (Figure 4B). Gene enrichment pathway analysis performed with GSEA supported this inflammatory signal and additionally demonstrated upregulated pathways in LS fibroblasts related to cell motility, proliferation, mesenchymal transition, and apoptotic response gene sets (Figure S3).

### 2.3. Predictive Interactions of Fibroblasts and Other Cells Using NicheNet Analysis

**Figure 5:**
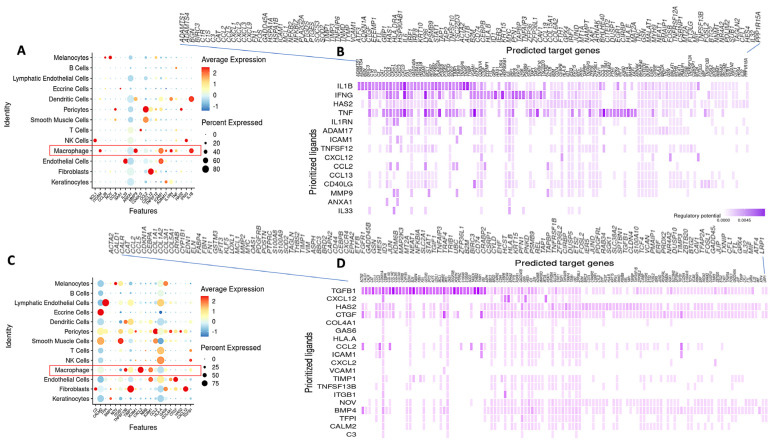
NicheNet analysis with fibroblast as receiver (**A**) displays inter/intracellular communication stemming from all cells (*y*-axis) to fibroblasts through top predicted ligands (*x*-axis). Macrophages have the highest influence on fibroblasts (red outlined box). (**B**) shows top predicted ligands (*y*-axis) sent from all cells and which genes (*x*-axis) these ligands interact with in fibroblasts (receiver). The most significant ligand in this subset was IL1B. (**C**) shows which cells fibroblasts interact with (*y*-axis) and which top ligands result in these interactions (*x*-axis), with fibroblasts set as the sender cell type. (**D**) displays top predicted ligands from fibroblasts (set as the sender cell type) to all other cell types, and the resulting regulated genes on the *x*-axis. The most significant ligand in this subset was TGFB1. Fibroblasts also strongly influence macrophages (red outlined box), therefore creating an auto-stimulatory cycle.

NicheNet was used to investigate intercellular communication between cells. Analyses were focused on the programming of fibroblasts as the receiver and sender cell type. The results revealed interesting communications between fibroblasts, macrophages, and T cells within our dataset. While investigating which cells have regulatory potential on fibroblasts, macrophages were found to have the highest influence on fibroblast gene expression through ligand interaction (Figure 5A). It was predicted that macrophages specifically communicate with fibroblasts through ligands IL1B, IFNG, TNFα, ITGAM, MMP9, ICAM1, IL1RN, and ADAM17 (Figure 5B). Notable communications were found stemming from ligand IL1B and influencing CXCL1-3,9 CCL2, and CCL5; ligand IFNG was found to be influencing CXCL9, ICAM1, GBP1, and HLA DRA; and ligand TNF demonstrated large, predicted influence on many genes including CCL19, COL1A1, COL6A2, and JUND. Fibroblasts are also predicted to influence the expression of CXCL12 in other fibroblasts.

Alternatively, fibroblasts set as the sender cell type strongly interact with macrophages through CXCL12, interacting with IL6, CXCL8, CD44, ID1, JUN, and CXCR4, creating an auto-stimulatory cycle between macrophages and fibroblasts (Figure 5C). While investigating the influence of fibroblasts on all other cells, high regulatory potential of COL1A1, COL1A2, COL3A2, and ACTA2 gene expression in cells (macrophages, T cells, and NK cells) through top-predicted ligand TGFB1 was determined (Figure 5C,D). Fibroblasts are also predicted to communicate with smooth muscle cells and pericytes through ligand CCL2, which interacts with genes COL1A1, ACTA2, and TGFB1 (Figure 5C,D). 

### 2.4. Cell-State Transition Using Monocle-Derived Pseudotime Analysis of Fibroblast Subclusters in LS and Healthy Skin Predicted a Transition in Clusters Containing Progenitors for Myofibroblasts

**Figure 6:**
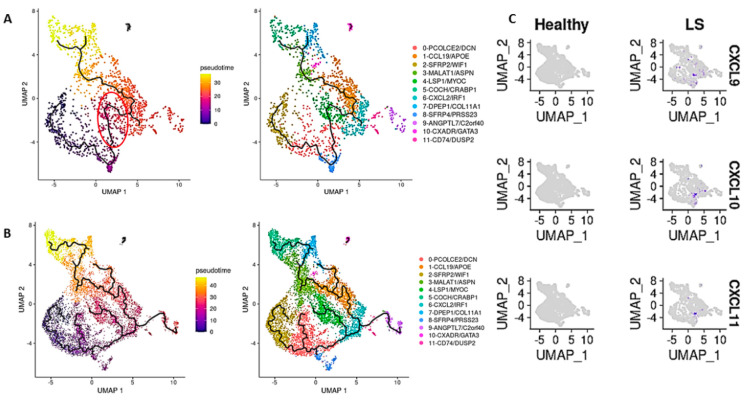
Pseudotime trajectory of fibroblasts comparing LS to healthy cells. (**A**) UMAP with a pseudotime trajectory projected on it with cells colored by pseudotime (left) and by subcluster (right) in LS fibroblasts. The trajectory traces through Cluster 8 cells connecting Cluster 0 to Cluster 4. In this transition zone from subcluster 8 to subcluster 4 there is a bridge only identified in LS and not HC which is circled in red. (**B**) UMAP with a pseudotime trajectory projected on it with cells colored by pseudotime (left) and by subcluster (right) in healthy cells. The trajectory does not trace through Cluster 8 cells at all, nor connects Cluster 0 to Cluster 4. (**C**) FeaturePlot showing the expression of CXCL9,10, and 11, signified by the purple dots, in fibroblasts (healthy on the left, LS on the right). Expression of CXCL9/10/11 coincides with the unique LS pseudotime trajectory through Cluster 0 (red circle in (**A**)), but are not expressed nor involved in the healthy pseudotime trajectory.

Pseudotime analysis was carried out on our dataset in order to make some inferences on potential cell-state transitions in fibroblasts [46] and was applied to total fibroblasts, LS fibroblasts, and healthy fibroblasts separately in order to observe differences in the cell-state transitions between the two groups. We observed that the trendline is different when comparing LS (Figure 6A) with healthy (Figure 6B). In LS, the trendline passes through the Cluster 8 (SFRP4/PRSS23) fibroblasts (myofibroblast-like cells), while in the healthy controls, the trendline does not pass through Cluster 8. We also observed that the LS trendline connecting Cluster 8 (SFRP4/PRSS23) to Cluster 4 (LSP1/MYOC) passes through a small subset of cells in Cluster 0 (PCOLCE2/DCN), like a “bridge” (Figure 6A), which upon investigation were highly expressive of cytokines and chemokines CXCL9/10/11, all known to be CXCR3 ligands (Figure 6C). We noted that this “bridge” area on the pseudotime map in Cluster 0 only expressed CXCL9/10/11 in LS patients and not in the healthy controls (Figure 6C), and that this passage was not involved in the healthy trendline (Figure 6B). 

## 3. Discussion

Abnormal fibroblast responses are central to the role of the development of fibrotic skin disease in both systemics sclerosis (SSc) and localized scleroderma (LS; morphea), with more recent understanding of likely pathogenic fibroblast subpopulations in SSc resulting from recent advances in scRNA seq technology [6,7,8,9,10]. However, this depth of understanding has not been fully explored in LS, with published studies of larger SSc cohorts that evaluate the cellular landscape using scRNA seq including only a few LS subjects without dedicated analyses [7]. Our group has previously demonstrated the importance of fibroblasts in playing a potential inflammatory role by showing fibroblasts co-localizing with T cells and macrophages [47,48] and the overlapping location in the dermis of fibrotic burden via collagen deposition and inflammatory infiltrate burden in the skin [48,49,50]. To our knowledge, we are the first to report a comprehensive study of the transcriptomic landscape of individual fibroblasts in LS skin (in children and adults) in an unbiased manner using scRNA seq. Our analyses provide an unprecedented view of fibroblast heterogeneity in LS, uncovering 12 populations with robust gene expression markers for each population and suggesting discrete functions for these fibroblast populations. We have also identified the following: three distinct clusters which are more prevalent in LS subjects compared with controls, one of which supports a myofibroblast-like identity; clusters that overlap in expression with identified SSc clusters, while others are unique to LS; an unexpected general inflammatory gene expression in LS fibroblasts (IFN- and HLA-related) compared with healthy fibroblasts; and potential roles for fibroblasts through cell–cell communication and trajectory software, especially macrophage interaction.

Our data show a global shift in fibroblast phenotypes in LS skin, with Cluster 1 (CCL19/APOE), Cluster 8 (SFRP4/PRSS23), and Cluster 10 (CXADR/GATA3) more predominantly expressed in diseased skin compared with control skin, with two of the three being similar to fibroblast populations of interest reported earlier in SSc skin studies (one with a myofibroblast-like phenotype, Cluster 8), while the third (Cluster 10) appears unique to LS, and all share an upregulation of CXCR3 related chemokines CXCL9/10/11 compared with healthy controls. 

Cluster 1 (CCL19/APOE) [8] and Cluster 8 (SFRP4/PRSS23) [8,9] both highly express genes that appear to have important roles in upregulation of wound healing and fibrosis [43,51]. CCL19/APOE has been identified as a major fibroblast subcluster in scRNA seq of SSc skin appearing mainly adjacent to vascular structures [9], with additional studies validating CCL19 expression in the skin of diffuse cutaneous SSc (dcSSc) patients using quantitative PCR analysis, finding its expression correlated with markers of vascular inflammation and macrophage recruitment [52]. CCL19 is also a chemo-attractant for macrophage and T cell recruitment, and this increased expression in the CCL19 subcluster observed in LS skin may suggest a role of CCL19 in the recruitment of immune cells, like macrophages and T cells, to the lesional site during the process of inflammation, making this disease similar in some ways to SSc. 

Cluster 8 (SFRP4/PRSS23) was identified as a myofibroblast-like cluster in our LS dataset based on our Scleroderma Center’s prior findings in SSc skin [9], with this population expressing shared upregulated genes SFRP2, PRSS23, SFRP4, ADAM12, and ACTA2 (Table S3). SFRP4 has also been associated with both skin and lung fibrosis, where it has been suggested to be a potential biomarker for SSc [53]. MFAP5 was also one of the most upregulated genes in this cluster and has earlier been found to be overly expressed in myofibroblasts [54] and identified as a marker for this cell type, since it is closest in gene expression to transitioning myofibroblasts found in skin and pulmonary fibroblasts in SSc patients [10]. Also highly expressed in the SFRP4/PRSS23 cluster, especially in LS, was FBNI, a protein-coding gene for the extracellular matrix component fibrillin-1. FBN1 variants are known to cause connective tissue disorders, including Marfan syndrome and stiff skin syndrome through alterations of the ECM. FBN1 expression is increased in scleroderma skin [55], which is unusually degradation-susceptible [56], and the presence of anti-fibrillin-1 antibodies in SSc is thought to activate fibroblasts and stimulate the release of TGF-β [57,58], all supportive of FBN1′s potential role in scleroderma pathogenesis. A higher proportion of these SFRP4/PRSS23 fibroblasts containing upregulated FBN1 and MFAP5 expression were demonstrated in the LS compared with the healthy control. Further regarding our SFRP4/PRSS23 cluster, an interesting observation was noted using pseudotime analysis, namely that when splitting the analysis into LS and healthy fibroblasts, the trajectory passes through Cluster 8 whereas healthy does not. The trendline in the LS fibroblasts then continues through an inflammatory CXCL9/10/11 predominate pathway towards other dermal cell populations, whereas when similar analysis was performed on healthy samples, there was no trendline in the trajectory that involved or passed through Cluster 8, which contains the population of myofibroblast-like cells (Figure 6). This demonstrates that the SFRP4/PRSS23 subcluster may be essential to LS disease propagation and will be the focus of future studies. 

Cluster 10 (CXADR/GATA3) was the third cluster more predominately expressed in LS compared with controls and highly expresses GATA3 in addition to genes found by GSEA to be associated with proliferation regulation, negative regulation of cell death, and epidermis development and differentiation. This is a fibroblast cluster that is unique to LS that we have identified. GATA3 expression was previously found in the skin of dcSSc patients, but in T cells [59,60]. GATA3 expression has been related to early inflammatory SSc disease and was found to be a novel potential therapeutic target in patients with SSc [61] due to its role in upregulating IL-13 synthesis in response to TGF-β. 

Eight of our 12 fibroblast subclusters (Clusters 0, 1, 2, 4, 5, 7, 8, 9) had similar expression and identification to those annotated in healthy and adult SSc skin by our Scleroderma Center (Lafyatis) (Table S3) [8,9]. These include clusters identified as dermal and papillary dermal, and dermal-sheath-associated fibroblasts. This highlights the reproducibility of fibroblast clusters of the skin with scRNA seq (healthy and disease), as well as shared pathophysiology between the two sclerodermatous disorders since potentially pathogenic fibroblasts, such as Cluster 8 (SFRP4/PRSS23), were identified in both conditions, SSc and LS. 

Unique fibroblast populations in LS include clusters 3 (MALAT/ASPN), 6 (CXCL2/IRF1), 10 (CXADR/GATA3) and 11 (CD74/DUSP2). The MALAT/ASPN fibroblast cluster was relatively large, but non-specific. The expression of ASPN has been documented in cells surrounding the hair follicle in the Human Atlas [62], so this population of fibroblasts may be related to hair follicles and/or papillary fibroblasts. A somewhat similar population was identified by our group in SSc (POSTN/ASPN) (Table S3) [8,9]. Cluster 11 (DUSP2/CD74) is a smaller subcluster of fibroblasts identified to be elevated in LS patients compared with controls (2.4% vs. 0.8%, respectively). This cluster is heavy in expression of both HLA-associated (HLA-DQB1, -DPA1, -DRB1, -DRA) and inflammatory gene expression (CXCR4, CD74, IL32) (Table S3).

Cluster 6 (CXCL2/IRF1) is a unique cluster identified in our study, with a higher proportion in LS subjects expressing CXCL, CCL, and interferon inflammatory gene expression profiles, with CXCL2 being the top upregulated gene in this cluster. High CXCL2 expression [43] is associated with neutrophil attraction, vascularization, and homing of bone-marrow-derived stem cells to wound sites during wound repair, and in tandem with high ICAM-1 expression in this cluster (Table S3), supports the role of possible endothelial interaction and neutrophil recruitment occurring in the environment of this fibroblast cluster. Future spatial studies of fibroblast locations relative to intradermal vessels are planned. Additional interrogation of the CXCL2/IRF1 fibroblast cluster identified high expression of IL-6 compared with other clusters (Figure S4). IL-6 is a cytokine known to increase the JUN mediated fibrotic pathways in cells [63], along with playing an important role in regulating cell proliferation, activation, and differentiation, with upregulation demonstrated in SSc [64]. Additionally, IL-6 plays a communicative role with M2 macrophages in wound healing [65]. IL-6 is known to regulate M2 macrophage polarization and is released by M1 macrophages at the wound site. M2 cells are important for late stage wound healing, but excess production of IL-6 can lead to overexpressed TGF-β, which activates collagen-1 expression in fibroblasts, facilitates ECM deposition, and inhibits ECM degradation [65].

The influence of macrophages on fibroblasts in LS was robustly demonstrated in our unsupervised NicheNet cell-communication analysis (Figure 5A,B). Macrophage ligands IL1-β, IFNγ, and TNF had the highest influence on fibroblast gene expression of CXCL and CCL ligands (via IL1-β), CXCL, HLA, and Interferon related genes (via IFNγ), and CCL19, JUND, and collagen related genes (via TNF), respectively. In turn, the overall influence of fibroblasts on all cells as a sender identified the expected collagen genes and ACTA2 expression through ligands TGFB1 and CTGF (Figure 5C,D). 

**Figure 7:**
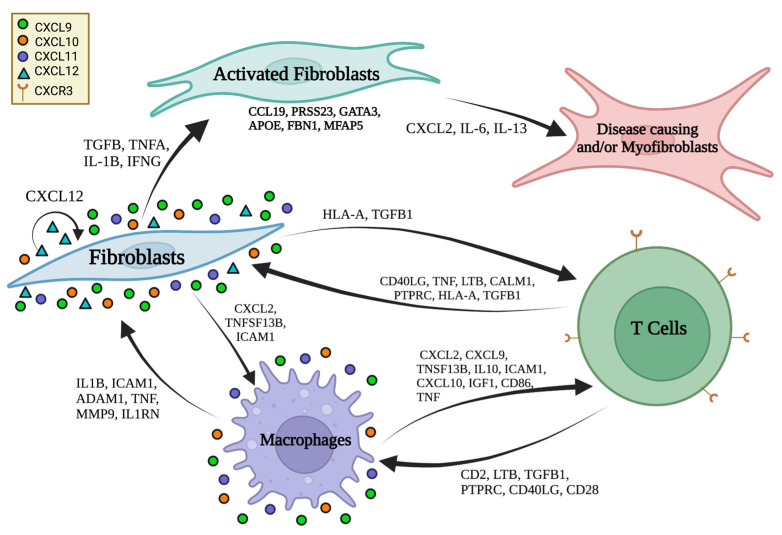
Proposed cellular interactions of macrophages, fibroblasts, and T cells in localized scleroderma. Resident macrophages directly influence the fibroblasts by producing inflammatory cytokines and chemokines (IL1B, IFN, TNF) to activate the fibroblasts to upregulate both inflammatory (CXCL12, CXCL9/10/11) and fibrotic gene (TGFB, CTGF) expression. This in turn leads to activated fibroblasts which auto-stimulate with CXCL12, enhance macrophage stimulation with CXCL2, and promote collagen-producing cells or diseased fibroblasts/myofibroblasts via CXCL9/10/11 stimulation, which are ligands for CXCR3 expressed on the T cells. It is likely that the T cells then migrate to the tissue and are responsible for influencing resident macrophages via inflammatory signatures which influence the macrophages to, in turn, activate the fibroblasts.

Since macrophages appear to have a critical role in stimulating fibroblasts, we conducted NicheNet analysis on macrophages as the receiver to further understand potential upstream influencers of macrophages. The results showed that the top interacting cell types with macrophages were dendritic cells, pericytes, smooth muscle cells, fibroblasts, endothelial cells, NK cells, and T cells (Figure S5A). From these sender cells, top predicted ligand IFNG was shown to have high regulatory potential on CCL2-5, CD14, CXCL8-11, and STAT1 expression in macrophages (Figure S5B). Top predicted ligand IL1B showed high regulatory potential on CXCL12, COL3A1, SOCS3, SOD2, STAT1, and CXCL8-11 expression (Figure S5B). TGFβ1, another top predicted ligand, showed influence on expression of CCL2,3,5 and CXCL10 in macrophages (Figure S5B). Overall, three quarters of the top four ligands (IFNG, IL1B, TNF, and TGFβ1) showed a strong impact on CXCL9/10/11, which are all CXCR3 ligands (Figure S5B). We also identified the motif of fibroblast to macrophage communication by ligand CXCL12 again (Figure S5B and Figure 5C,D). Linking these cell communication data together, we gather that T cells impact macrophage expression of both inflammatory and profibrotic gene expression, and these macrophages in turn heavily influence inflammatory and collagen expression in fibroblasts, leading to activated fibroblasts which may be pathogenic (Figure 7).

In addition to this communication pattern between cell types, we also demonstrated a strong inflammatory cytokine signal comprising CXCL9/10/11, known ligands for CXCR3, throughout our data from the LS single-cell analyses methodologies, including a robust expression of these ligands in LS compared with healthy controls, as a predominate influencer in cell-communication analyses, and included in the trajectory line in pseudotime only in the LS model and not in the healthy controls. This strong CXCL9/10/11 inflammatory signature influence supports our collective group’s prior work in LS (morphea) (Torok and Jacobe), including identification of CXCR3 ligand predominate expression in microarray skin [66], RNA bulk seq skin [67,68], and IHC skin [47,48], with close approximation of macrophages expressing CXCL9/10 and T cells (CD3/CD4) both adjacent to fibroblasts [47,48], and in peripheral blood via circulating cytokine [48,69,70] and flow cytometry [47] findings in LS subjects. The CXCR3 ligand signature (CXCL9/10/11) often correlated with validated measures of both disease activity, clinical measures and fibrotic load (collagen deposition) in LS patients [48,68,69,70]. Furthermore, we have demonstrated that CXCL9, and to a lesser extent CXCL10, along with receptor CXCR3, are necessary for the development of skin fibrosis in a morphea mouse model (bleomycin mouse with limited days of bleomycin exposure) [71]. Collectively, our current study and prior data support a strong CXCL9/10/11 influence in LS, providing possible therapeutic targets and evidence that LS is an inflammatory driven fibrotic disease.

### Limitations

Despite the promising observations, a more detailed insight is required to be able to define the pathogenesis and progression of this disease in LS patients. Our scRNA-seq analysis was limited by a relatively small sample size per LS subtype. We aim to increase our cohort size and investigate if there are fibroblast subcluster and gene expression differences between subtype (linear trunk, linear head, generalized morphea, etc.) as well as between pediatric- and adult-onset LS. This is also a cross-sectional study design, and changes in fibroblast subcluster proportion and gene expression over time as the disease improves or worsens will provide valuable information. 

## 4. Methods

### 4.1. Human-Patient Skin Samples

Skin samples were obtained from research participants in the National Registry of Childhood Onset Scleroderma (NRCOS) (University of Pittsburgh #PRO11060222), Connective Tissue Disease (CTD) Registry (University of Pittsburgh, #PRO19090054), and Morphea in Adults and Children (MAC) Registry (University of Texas Southwestern, #STU112010-028) cohorts. LS patients enrolled all met the Padua diagnostic criteria for LS [72] and were categorized as pediatric onset if disease onset of < 18 years of age and adult onset if onset ≥18 years of age. Healthy controls were taken from tissue discard IRB (University of Pittsburgh, #STU19070023) and were age- and sex-matched. Two 4 mm punch biopsies of the affected area were taken at the time of collection (affected area for LS), one allocated for scRNA seq and the other for paraffin embedding. The average depth of the biopsies was 3.4 mm.

### 4.2. Single-Cell RNA Sequencing 

Samples were either processed immediately (fresh) or preserved in CryoStor^®^ CS10 preservation media (BioLife Solutions^®^, Bothell, WA, USA) (frozen), as described in our earlier feasibility publication [25]. Samples were enzymatically digested (Miltenyi Biotec Whole Skin Dissociation Kit, human Cat# 130-101-540 Miltenyi Biotec^®^, Gaithersburg, MD, USA) for 2 hours and further dispersed using the Miltenyi gentleMACS Octo Dissociator (Cat# 130-096-427, Miltenyi Biotec^®^, Gaithersburg, MD, USA). The resulting cell suspension was filtered through 70-μm cell strainers twice and re-suspended in PBS containing 0.04% BSA. Cell suspensions were then mixed with reverse transcription reagents and loaded into the Chromium instrument (10x Genomics^®^, Pleasanton, CA, USA), a commercial application of Drop-Seq [73]. The Chromium instrument then formed GEMs, which are gel bead-in-emulsions. GEMs contain a gel bead, scaffold for an oligonucleotide that is composed of an oligo-dT section for priming reverse transcription, and barcodes for each cell (10x Genomics^®^) and each transcript (unique molecular identifier, UMI) [74]. This instrument separated cells into mini-reaction “partitions” formed by oil microdroplets, each containing a gel bead and a cell. Approximately 1000-fold excess of partitions compared to cells ensured low capture of duplicate cells. Approximately 2600–4300 cells were loaded into the instrument to obtain data on ~1100–2300 cells, anticipating a multiplet rate of ~1.2% of partitions. V1 and V2 single-cell chemistries were used in accordance with the manufacturer’s protocol (10x Genomics).

The reaction mixture/emulsion was briefly removed from the Chromium instrument, and reverse transcription was performed. The emulsion was then broken using a recovery agent, and following Dynabead and SPRI clean up, cDNAs were amplified by 11–12 cycles of PCR (C1000, Bio-Rad, Hercules, CA, USA). cDNAs were sheared (Covaris, Woburn, MA, USA) into ~200 bp length. DNA fragment ends were repaired and A-tailed and adaptors were ligated. The library was quantified using a KAPA Universal Library Quantification Kit KK4824 and further characterized for cDNA length on a bioanalyzer using a high-sensitivity DNA kit. Libraries were sequenced (~200 million reads/sample), using the Illumina NextSeq-500 platform.

### 4.3. Data Preprocessing and Bioinformatics Analyses

Sequencing reads were examined by quality metrics and transcripts were mapped to reference human genome (GRCh38). Cell Ranger (10x Genomics) was used to assign reads to particular cells according to their barcode. Data from the study will be deposited on NCBI Gene Expression Omnibus (GSE number to be determined, pending).

Data analyses were performed using R (version 4.2), specifically the Seurat 4.3 package for normalization of gene expression and identification and visualization of cell populations [75,76]. Principal component analysis (PCA) was performed on the highly variable genes, and the Harmony [77] package was used to integrate the dataset, removing variation by sample (library_id) before cells were clustered using a smart local moving algorithm (SLM) [78] and visualized by UMAP [79]. AddModuleScore was utilized to calculate the average expression levels of each input (either gene or cluster) on a single-cell level, subtracted by the aggregated expression of control feature sets to define cell clusters. 

Differential gene expression was performed between sample types, LS compared with healthy, among the cell clusters and subclusters using the Libra [80] package’s edgeR based “pseudobulk” methodology. *p*-values were calculated and adjusted according to the Benjamini Hochberg method, and differential expression was reported as a log-fold change (log_FC). The package EnhancedVolcano was used to visualize differentially expressed genes through volcano plots.

Pathway analysis was then performed on the genes upregulated in LS compared to healthy using Reactome [81], which utilizes a relational database of signaling and metabolic molecules to compute and organize biological pathways. *p*-values were generated from over-expression analysis.

### 4.4. Cell-Communication Analysis 

Analysis between the main cell types (i.e., fibroblasts, macrophages, T cells) was investigated using NicheNet [82]. NicheNet is a package that utilizes ligand-target interaction matrices, gene regulatory interactions, as well as signal transduction in order to make predictions based on our input Seurat object [82]. 

### 4.5. Pseudotime Analysis 

Pseudotime analysis was carried out on our dataset in order to make some inferences on potential cell-state transitions in fibroblasts [46]. Monocle 3 was used to construct “pseudotime” single-cell trajectories [83]. SeuratWrappers was used to convert our Seurat object to a Monocle object. Cells were clustered using Louvain/Leiden community detection and nonlinear dimensionality reduction was performed using UMAP. The reversed graph embedding machine-learning technique was used to construct pseudotime trajectories [84]. 

## 5. Conclusions

In this scRNA-seq study, we show the heterogenous nature of fibroblasts in the skin of localized scleroderma (morphea) patients, with approximately two thirds of the subclusters with similar expression profiles seen in prior healthy control and systemic sclerosis (SSc) scRNA-seq studies, including a myofibroblast-like population (SFRP4/PRSS23) with features shared with SSc, while a few unique subclusters were identified in this LS study, with distinct inflammatory gene signatures (CXCL, CCL, HLA). 

One of the new clusters identified, Cluster 6 (CXCL2/IRF1), is of particular interest due to its relatively unique expression of IL-6 in LS cells, linked with known IL-6 involvement in the promotion of macrophage–fibroblast communication in fibrosis, and our NicheNet findings of strong influence of macrophages on fibroblasts promoting both inflammatory and fibrotic expression in fibroblasts. Tocilizumab, an IL-6 inhibitor, has been FDA approved for the treatment of interstitial lung disease in SSc, especially effective in the inflammatory phase of disease (patients with high C-reactive protein) [85]. Our data supports such an IL-6 inhibitor possibly being effective in LS, which has been observed to be effective in case reports of refractory LS [86]. Further investigation into IL-6 inhibition in LS is warranted, as a therapeutic agent is readily available. 

Another target of interest derived from this study and supported in our prior work would be the CXCR-3 related chemokines, CXCL9/10/11. These chemokines are significantly elevated in most fibroblast populations in LS compared with healthy controls and are important gene targets in fibroblasts influenced by macrophages in cell-communication analyses; furthermore, a CXCL9/10/11 conduit seemed essential for the trajectory of the LS pseudotime cell-transition analyses, directly after the path crossed through the myofibroblast-like population (Cluster 8). Janus kinase inhibitors (JAK inhibitors) are not necessarily specific, but have been shown to significantly decrease CXCR-3 related chemokines in humans and mouse models [71,87,88,89]. Case reports of JAK inhibitors in LS (morphea) support their use as a therapeutic agent [90]. Future investigation is deserved in this area. Combined with our cell-communication data, this study suggests that applying medications such as JAK inhibitors and IL-6 inhibitors may be most effective in the early stages of LS—when macrophages (indirectly T cells) are influencing fibroblasts to activate inflammatory and fibrotic gene expression—potentially inhibiting a large amount of collagen deposition and fibrosis in the dermis and deeper connective tissue, highly attenuating the impact of the disease, and improving the quality of life in patients with LS.

## Figures and Tables

**Table 1:** Demographic and clinical variables—LS subjects (full details Table S1).

Attributes	LS Patients (*n* = 14)
Gender, Female, *n* (%)	8 (57%)
Age at time of biopsy (years), mean (SD)	38.29 (22.50)
Pediatric Age at time of biopsy (years), mean (SD)	15.25 (5.05)
Adult Age at time of biopsy (years), mean (SD)	55.38 (12.11)
Pediatric Age at disease onset (years), mean (SD)	10.1 (4.60)
Disease duration (months), mean (SD)	10.53 (6.20)
Ethnicity, *n* (%) (Non-Hispanic)	13 (92.8)
Race, n (%)	
Caucasian	11 (78.6)
Asian	2 (14.3)
Hispanic	1 (7.14)
Disease Subtype, n (%)	
Linear trunk/limb	5 (35.71)
Linear face/scalp	2 (14.29)
Circumscribed morphea	2 (14.29)
Generalized morphea	5 (35.71)
**Clinical Disease Features**, median (IQR)	Active (*n* = 11)/ Inactive (*n* = 3)
Pediatric mLoSSI	7.33 (6.47)
Adult mLoSSI	14.5 (19.37)
PGA-A	27.1 (28.7)

## Data Availability

Data from the study will be deposited on NCBI Gene Expression Omnibus as fastq files (raw) and processed data.

## Supplementary Materials

The supporting information can be downloaded at: https://www.mdpi.com/article/10.3390/ijms24129796/s1.
