# Supplementary material for: Single-Cell Transcriptome Analysis Identifies Subclusters with Inflammatory Fibroblast Responses in Localized Scleroderma
Source: Int J Mol Sci. 2023 Jun 6;24(12):9796. doi: 10.3390/ijms24129796 (PMC10298454; doi:10.3390/ijms24129796)
Supplement: Supplementary file 1 [file ijms-24-09796-s001.zip › Supplemental figure legends.pdf]

SUPPLEMENTARY FIGURES

Figure S1: A) A total of 60,966 cells (32,154 healthy and 28,812 LS) clustered into 43 clusters before annotation. Each dot represents an individual cell on the UMAP plot. B) An even disbursement of healthy and LS cells throughout the main cellular groupings after annotation. C) Annotations from the literature used to pair down clusters into 14 main groups. D) Fibroblasts identified in upper right hand corner with a feature plot for a combination of COL1A1, COL1A2, and PDGFRA gene expression

Figure S2: Dot plot of gene expression and percent expressed per subcluster, with main two genes determining cluster in both LS and Healthy cells.

Figure S3: Top 20 GSEA indicated functions for upregulated genes. Gene enrichment pathway analysis performed on upregulated LS genes with GSEA demonstrates an inflammatory signal and upregulated pathways related to cell motility, proliferation, mesenchymal transition, and apoptotic response gene sets in LS fibroblasts.

Figure S4: Violin plot of IL6 in all clusters of fibroblasts split amongst LS and healthy individuals with cluster 0, cluster 6 and cluster 8 showing a high upregulation in IL6 in the LS patients compared to control. Cluster 6 (CXCL2/IRF1) had the highest IL6 expression, specifically in LS patients.

Figure S5: Nichnet analyses focused on macrophages as receiver. Figure A) displays the intercellular communication sent from all cell types to the receiver cell type macrophages. Fibroblasts influence macrophages through CXCL12. Dendritic cells, endothelial cells and others also influence macrophages through various inflammatory gene expression. Figure B) displays the top predicted ligands received by the macrophages from other cells on the y-axis, and what genes they communicate within the macrophage on the x-axis. The top predicted ligand IFNG was the highest influencer with high regulatory potential on CCL2-5, CD14, CXCL8-11 and STAT1 expression.
