# Supplementary material for: Single-Cell Transcriptome Analysis Identifies Subclusters with Inflammatory Fibroblast Responses in Localized Scleroderma
Source: Int J Mol Sci. 2023 Jun 6;24(12):9796. doi: 10.3390/ijms24129796 (PMC10298454; doi:10.3390/ijms24129796)
Supplement: Supplementary file 1 [file ijms-24-09796-s001.zip › Supplementary table legends.pdf]

SUPPLEMENTARY TABLES

Table A1: Table containing all clinical and biopsy data pertaining to the 14 healthy and 14 LS patients within this study. The top 14 rows contain data relevant to the affected patients and the bottom 14 contain information relevant to the healthy controls.

Table A2: Table containing cell counts from each of the 14 cell types for all 28 samples along with totals and percent of each cell type for healthy and LS patients.

Table A3: This table displays the top 20 differentially expressed genes for each cluster of the 12 fibroblast clusters in our dataset (LS and Healthy control). Genes bolded in text indicate that they were also found in the SSc fibroblasts (and healthy control) as seen in adult scleroderma studies at our scleroderma center headed by Robert Lafyatis (Tabib et.al 2021)*. Not only were many of the same genes found, many clusters matched with a high margin of genes. Our cluster 0-POLCE2/DCN matched their cluster 1-POLCE2, with 13 of our top 20 DEGS in their list as well. Our cluster 1-CCL19/APOE matched significantly with Lafyatis' cluster 0-CCL19/APOE, with 14/20 of our genes in their top 20. Our cluster 2-SFRP/WIF1 was extremely similar to their cluster 3-SFRP2/WIF1, with all 20 of our top 20 DEGs in this cluster found in their top 20 DEG dataset. A few of these genes were found in their dataset in other clusters, which were COMP, COL1A1, STC2, COL3A1 and PRSS23. Our cluster 4-LSP/MYOC shared 16/20 of the same genes with their cluster 6-MYOC/FMO1/APOE. Our cluster 5-COCH/CRABP1 showed 17/20 of our genes were also found in their cluster 5-CRABP1. Our cluster 7-DPEP1/COL11A1 had 13/20 of the same genes as their cluster 8-COL11A1. Finally, our cluster 9-ANGPTL7/C2orf40 shared 12/20 genes with their cluster 7-ANGPLT7. Other matches were found within clusters with only a few similar genes. Overall, 115/240 genes found in our top 20 DEG dataset were also found in adult SSc top 20 DEG dataset. Asterisk next to LS cluster name signify analogous cluster to the SSc/healthy dataset. Notable are the four uniquely identified clusters in this LS/healthy dataset; clusters 3 (MALAT/ASPN), 6 (CXCL2/IRF1), 10 (CXADR/GATA3) and 11 (CD74/DUSP2).

*(Tabib et.al 2021)- T. Tabib *et al.*, "Myofibroblast transcriptome indicates SFRP2(hi) fibroblast progenitors in systemic sclerosis skin," *Nat Commun,* vol. 12, no. 1, p. 4384, Jul 19 2021, doi: 10.1038/s41467-021-24607-6

Table A4: This table contains summary information regarding the 9192 fibroblast cells found in our dataset. Here we are comparing healthy and LS cells for each of the 12 clusters. Included are the total counts of fibroblasts, total counts of healthy fibroblasts and total count of LS fibroblasts.
