# Supplementary material for: Single-Cell Transcriptome Analysis Identifies Subclusters with Inflammatory Fibroblast Responses in Localized Scleroderma
Source: Int J Mol Sci. 2023 Jun 6;24(12):9796. doi: 10.3390/ijms24129796 (PMC10298454; doi:10.3390/ijms24129796)
Supplement: Supplementary file 1 [file ijms-24-09796-s001.zip › Supplementary Table S1 Demographic table.pdf]

Sample ID	Chemistry	Age (yrs)	Gender	Race/Ethnicity	Sample Type	Active/Inactive	Onset	Age of onset	Disease Duration	Antibody Status	Subtype	LoSAI/ mLoSSI	LoSDI	PGA-A	PGA-D	PGA-S	Bx Location
**SC266**	V2	37	M	Caucasian	Cryostor	A	Adult	24	13	unknown	Linear	4	34	13	55	-	Abdomen
**SC267**	V2	64	F	Caucasian	Cryostor	A	Adult	61	2	unknown	Generalized	9	14	23	12	-	Left abdomen
**SC272**	V2	43	F	Hispanic	Cryostor	A	Adult	14	9	unknown	Linear	10	10	23	15	-	Left abdomen
**SC275**	V2	65	F	Caucasian	Cryostor	A	Adult	63	2	unknown	Generalized, Isomorphic morphea	5	32	10	23	-	Left flank
**SC222AC**	V2	65	M	Caucasian	Affected Cryostor	A/I	Adult	-	-	unknown	Generalized	14	38	18	18	-	Left abdomen
**SC260**	V2	43	M	Caucasian	Fresh	I	Adult	34	13	unknown	Circumscribed/ Plaque	0	5	0	18	12	Lower back
**HJ809**	V2	65	F	Caucasian	Cryostor	A	Adult	64	1	unknown	Generalized	61	28	90	60	-	Abdomen
**HJ867**	V2	61	F	Caucasian	Cryostor	A	Adult	54	8	unknown	Generalized	13	39	27	30	-	Right Arm
**SC126**	V2	8	F	Caucasian	Fresh	A	Peds	6.3	9.19	ANA negative, Histone negative, ssDNA negative	Linear	17	14	76	35	43	Upper thigh
**SC198**	V2	14	F	Asian	Fresh	I	Peds	13	14.69	unknown	Linear	0	8	0	18	19	Midarm
**SC246**	V2	15	F	Asian	Fresh	A	Peds	12.6	13.97	ANA positive (1:160 homogenous), Histone+ ssDNA+	Linear	13	12	62	37	47	Thigh
**SC259**	V2	16	M	Caucasian	Fresh	A	Peds	7.4	15.85	6/2019: Histone negative, ssDNA negative	Linear Face	4	4	29	41	45	Scalp/forehead
**SC414**	V2	20	M	Caucasian	Fresh	I	Peds	16.6	20.56	not done at this visit	Circumscribed	7	14	0	52	53	Lower back
**SC300**	V2	20	M	Caucasian	Fresh	A	Peds	4.8	17.16	not done at this visit	Linear Face	3	5	8	47	52	Scalp/forehead
**SC32**	V1	23	F	Asian	Cryostor	N/A	Peds	N/A	N/A	N/A	Healthy	N/A	N/A	N/A	N/A	N/A	Forearm
**SC296**	V2	5	M	Caucasian	Cryostor	N/A	Peds	N/A	N/A	N/A	Healthy	N/A	N/A	N/A	N/A	N/A	Scalp
**SC297**	V2	14	F	Hispanic	Cryostor	N/A	Peds	N/A	N/A	N/A	Healthy	N/A	N/A	N/A	N/A	N/A	Midarm
**HSK053**	V2	18	F	African American/ Caucasian	Fresh	N/A	Peds	N/A	N/A	N/A	Healthy	N/A	N/A	N/A	N/A	N/A	Breast
**HSK054**	V2	17	F	African American	Fresh	N/A	Peds	N/A	N/A	N/A	Healthy	N/A	N/A	N/A	N/A	N/A	Breast
**PHC003**	V2	13	F	Hispanic	Cryostor	N/A	Peds	N/A	N/A	N/A	Healthy	N/A	N/A	N/A	N/A	N/A	Midarm
**SC50**	V2	64	M	Caucasian	Fresh	N/A	Adult	N/A	N/A	N/A	Healthy	N/A	N/A	N/A	N/A	N/A	Forearm
**SC68**	V2	48	F	Caucasian	Fresh	N/A	Adult	N/A	N/A	N/A	Healthy	N/A	N/A	N/A	N/A	N/A	Forearm
**SC124**	V2	54	M	Caucasian	Fresh	N/A	Adult	N/A	N/A	N/A	Healthy	N/A	N/A	N/A	N/A	N/A	Forearm
**SC125**	V2	61	M	African American	Fresh	N/A	Adult	N/A	N/A	N/A	Healthy	N/A	N/A	N/A	N/A	N/A	Forearm
**SC1**	V1	63	M	Caucasian	Fresh	N/A	Adult	N/A	N/A	N/A	Healthy	N/A	N/A	N/A	N/A	N/A	Forearm
**SC4**	V1	63	M	Caucasian	Fresh	N/A	Adult	N/A	N/A	N/A	Healthy	N/A	N/A	N/A	N/A	N/A	Forearm
**SC18**	V1	66	F	Caucasian	Fresh	N/A	Adult	N/A	N/A	N/A	Healthy	N/A	N/A	N/A	N/A	N/A	Forearm
SC33	V1	62	F	Caucasian	Fresh	N/A	Adult	N/A	N/A	N/A	Healthy	N/A	N/A	N/A	N/A	N/A	Forearm
