# Supplementary material for: Single-Cell Transcriptome Analysis Identifies Subclusters with Inflammatory Fibroblast Responses in Localized Scleroderma
Source: Int J Mol Sci. 2023 Jun 6;24(12):9796. doi: 10.3390/ijms24129796 (PMC10298454; doi:10.3390/ijms24129796)
Supplement: Supplementary file 1 [file ijms-24-09796-s001.zip › Supplementary Table S2 all cells.pdf]

	Healthy														LS																		
**Cell Type**	**HSK053**	**HSK054**	**PHC003**	**SC1**	**SC124**	**SC125**	**SC18**	**SC296**	**SC297**	**SC32**	**SC33**	**SC4**	**SC50**	**SC68**	**HJ809**	**HJ867**	**SC126**	**SC144**	**SC198**	**SC222**	**SC246**	**SC259**	**SC260**	**SC266**	**SC267**	**SC272**	**SC275**	**SC300**	**Healthy**	**LS**	**Total**	**% Healthy**	**%LS**
Keratinocytes	1891	555	141	395	1016	759	981	236	143	136	148	414	404	172	156	62	2064	3139	1111	120	2780	898	2070	144	157	71	123	977	7391	13872	21263	34.8%	65.2%
Fibroblasts	338	806	69	301	764	448	911	104	211	784	999	501	382	732	38	66	85	140	364	128	186	205	83	51	68	104	50	274	7350	1842	9192	80.0%	20.0%
Endothelial Cells	338	523	517	83	468	184	137	299	497	146	103	502	486	135	663	528	195	282	124	413	302	162	185	602	322	502	617	286	4418	5183	9601	46.0%	54.0%
Macrophage	148	511	13	28	52	35	107	19	24	52	109	34	32	38	127	24	51	92	118	489	155	20	8	9	37	31	7	35	1202	1203	2405	50.0%	50.0%
NK Cells	65	81	17	12	24	31	53	32	33	62	72	8	18	26	332	35	135	213	349	312	217	60	13	27	26	75	37	28	534	1859	2393	22.3%	77.7%
T Cells	161	167	32	63	68	39	94	105	33	156	146	40	39	36	435	14	145	315	440	484	129	119	34	24	70	180	38	163	1179	2590	3769	31.3%	68.7%
Smooth Muscle Cells	165	78	83	87	326	312	263	86	139	161	107	333	609	231	169	100	105	84	112	89	89	272	97	520	119	140	201	135	2980	2232	5212	57.2%	42.8%
Pericytes	258	96	41	44	182	196	80	131	123	71	48	169	244	57	84	52	61	32	117	37	80	68	58	224	102	219	105	86	1740	1325	3065	56.8%	43.2%
Dendritic Cells	136	94	11	20	37	24	54	23	14	43	71	19	23	23	61	5	29	61	70	111	51	23	9	8	23	15	6	31	592	503	1095	54.1%	45.9%
Eccrine Cells	22	41	81	12	79	50	20	35	24	58	35	121	113	38	8	4	25	27	10	5	32	72	41	125	94	34	3	71	729	551	1280	57.0%	43.0%
Lymphatic Endothelial Cells	65	30	4	15	4	14	12	27	28	9	6	15	16	16	97	8	37	36	10	25	10	10	9	20	22	24	22	16	261	346	607	43.0%	57.0%
B Cells	27	11	4	2	5	1	6	2	2	6	44	1	1	3	66	6	9	13	251	10	8	8	3	6	2	1	2	5	115	390	505	22.8%	77.2%
Melanocytes	5	12	1	14	33	4	31	7	10	16	5	22	8	14	0	1	21	24	7	1	7	13	23	11	4	0	1	50	182	163	345	52.8%	47.2%
Mast Cells	4	3	0	2	39	9	7	0	0	9	10	5	8	43	3	0	40	28	2	0	3	1	2	0	0	1	0	15	139	95	234	59.4%	40.6%
**Totals:**	3623	3008	1014	1078	3097	2106	2756	1106	1281	1709	1903	2184	2383	1564	2239	905	3002	4486	3085	2224	4049	1931	2635	1771	1046	1397	1212	2172	28812	32154	60966		
