# Supplementary material for: Single-Cell Transcriptome Analysis Identifies Subclusters with Inflammatory Fibroblast Responses in Localized Scleroderma
Source: Int J Mol Sci. 2023 Jun 6;24(12):9796. doi: 10.3390/ijms24129796 (PMC10298454; doi:10.3390/ijms24129796)
Supplement: Supplementary file 1 [file ijms-24-09796-s001.zip › Supplementary Table S3 fibro all clusters and comparism.pdf]

0-POLCE2/DCN*	1-CCL19/APOE*	2- SFRP2/WIF1*	3-MALAT1/ASPN	4-LSP/ MYOC*	5-COCH/CRABP1*	6-CXCL2/IRF1	7-DPEP1/COL11A1*	8-SFRP4/ PRSS23	9-ANGPTL7/c2orf40*	10-CXADR/GATA3	11-CD74/DUSP2
**PI16**	**CCL19**	**COMP**	MALAT1	**ITM2A**	**COCH**	CXCL2	**DPEP1**	**SFRP4**	**ANGPTL7**	CXADR	CD37
**WISP2**	**APOE**	**WIF1**	MT-CYB	**MYOC**	**ASPN**	NFKBIA	**COL11A1**	LINC01133	**C2orf40**	EHF	CD52
**FBLN1**	**CXCL12**	**APCDD1**	MT-CO3	**GPC3**	**TNN**	IRF1	**PPP1R14A**	PRG4	**ITGA6**	IRF6	CORO1A
C1QTNF3	**RBP5**	**COL18A1**	MT-CO2	**GSN**	**CRABP1**	CSRNP1	**EDNRA**	SULF1	COL9A3	GATA3	LAPTM5
**DCN**	**IGFBP7**	**SFRP2**	MT-ND4	AADAC	**SLITRK6**	ATF3	**WFDC1**	HSD3B7	TENM2	LAD1	SRGN
**SLPI**	**RARRES2**	**STC2**	**MT-ATP6**	**EFEMP1**	SLC22A16	SOCS3	**TNMD**	**PRSS23**	FOXS1	DSC3	DUSP2
**SFRP2**	**TNFSF13B**	**HSPB3**	MT-ND1	**MGP**	**MKX**	ICAM1	**POSTN**	TRAC	EBF2	MPZL2	CXCR4
**CD55**	**B2M**	**COL6A1**	**MT-CO1**	**CYGB**	RSPO4	EGR1	**GPC3**	SERPINE1	**SCN7A**	CDH1	GMFG
QPCT	**CD74**	**CLEC2A**	MT-ND2	**APOD**	**NDNF**	JUNB	**MEF2C**	ACOT7	**SBSPON**	KLC3	HLA-DQB1
**ANGPTL5**	**CTSH**	**NKD2**	MT-ND5	**CFD**	**PLPP5**	MYC	**PRSS23**	SPHK1	**SLC22A3**	FGFR3	CREM
MMP2	**HLA-B**	**CD9**	**ASPN**	**IGFBP3**	**FIBIN**	GEM	DKK3	TMSB4X	**TM4SF1**	PKP3	HLA-DPA1
**CTHRC1**	RPS18	**LGALS1**	ADD3	**CXCL12**	**SPARCL1**	CDKN1A	**TNN**	PPP1R14B	**NGFR**	DSG3	BIRC3
**CLU**	TMEM176B	**COL6A2**	**NOVA1**	**GPX3**	EMID1	PPP1R15A	**RPS3A**	SEMA3C	**SLC2A1**	TINCR	HLA-DRB1
**MFAP5**	PTGDS	**NBL1**	SCAF11	**GGT5**	**TNMD**	ZFP36	**F2R**	FBN1	ENTPD2	PLPP2	ARHGDIB
**PCOLCE2**	**C3**	**TGFBI**	MPHOSPH8	**GDF10**	**PCSK1N**	SOD2	RPS2	SH3BGRL3	**SFRP4**	PKP1	**CD74**
**CPE**	**GGT5**	**COL1A1**	LIMA1	MGST1	**FZD1**	NFKBIZ	RPS3	TNFRSF12A	CAVIN2	KLK11	VPS37B
FBN1	**IFITM3**	**COL13A1**	RPS8	**IGFBP6**	**GPM6B**	ERRFI1	RPL10A	**MFAP5**	ITGB4	CDHR1	IL32
DPP4	RPS19	**RGCC**	HMGN1	LSP1	**HSPA2**	MAFF	RPL5	LOXL2	CLDN1	SERPINB5	HLA-DRA
FAM180B	RPL13	**LEPR**	UBE2B	FMO1	**OGN**	IER3	LAMC3	LOXL1	**KLF5**	SYTL1	**A2M**
PDGFRL	RPS27A	**COL3A1**	**RPL31**	**APOC1**	**PLXDC1**	CCL2	EEF1A1	LTBP2	**NR2F2**	DSP	STK4
