# Supplementary material for: Single-Cell Transcriptome Analysis Identifies Subclusters with Inflammatory Fibroblast Responses in Localized Scleroderma
Source: Int J Mol Sci. 2023 Jun 6;24(12):9796. doi: 10.3390/ijms24129796 (PMC10298454; doi:10.3390/ijms24129796)
Supplement: Supplementary file 1 [file ijms-24-09796-s001.zip › Supplementary Table S4 fibroblast all cluster cell percentage.pdf]

	Healthy	LS	Healthy (count)	LS (count)
**0-PCOLCE2/DCN**	18.26%	10.9%	1342	200
**1-CCL19/APOE**	13.77%	21.9%	1012	404
**2-SFRP2/WIF1**	14.80%	12.6%	1088	233
**3-MALAT1/ASPN**	13.03%	7.4%	958	136
**4-LSP1/MYOC**	10.01%	9.5%	736	175
**5-COCH/CRABP1**	9.12%	5.9%	670	108
**6-CXCL2/IRF1**	7.93%	10.2%	583	187
**7-DPEP1/COL11A1**	5.47%	7.2%	402	133
**8-SFRP4/PRSS23**	2.48%	6.2%	182	114
**9-C2orf40/ANGPTL7**	3.03%	2.8%	223	52
**10-CXADR/GATA3**	1.32%	2.99%	97	55
**11-CD74/DUSP2**	0.76%	2.44%	57	45
**Total**			**7350**	**1842**
