# Supplementary figures and images for: Single-Cell Transcriptome Analysis Identifies Subclusters with Inflammatory Fibroblast Responses in Localized Scleroderma
Source: Int J Mol Sci. 2023 Jun 6;24(12):9796. doi: 10.3390/ijms24129796 (PMC10298454; doi:10.3390/ijms24129796)

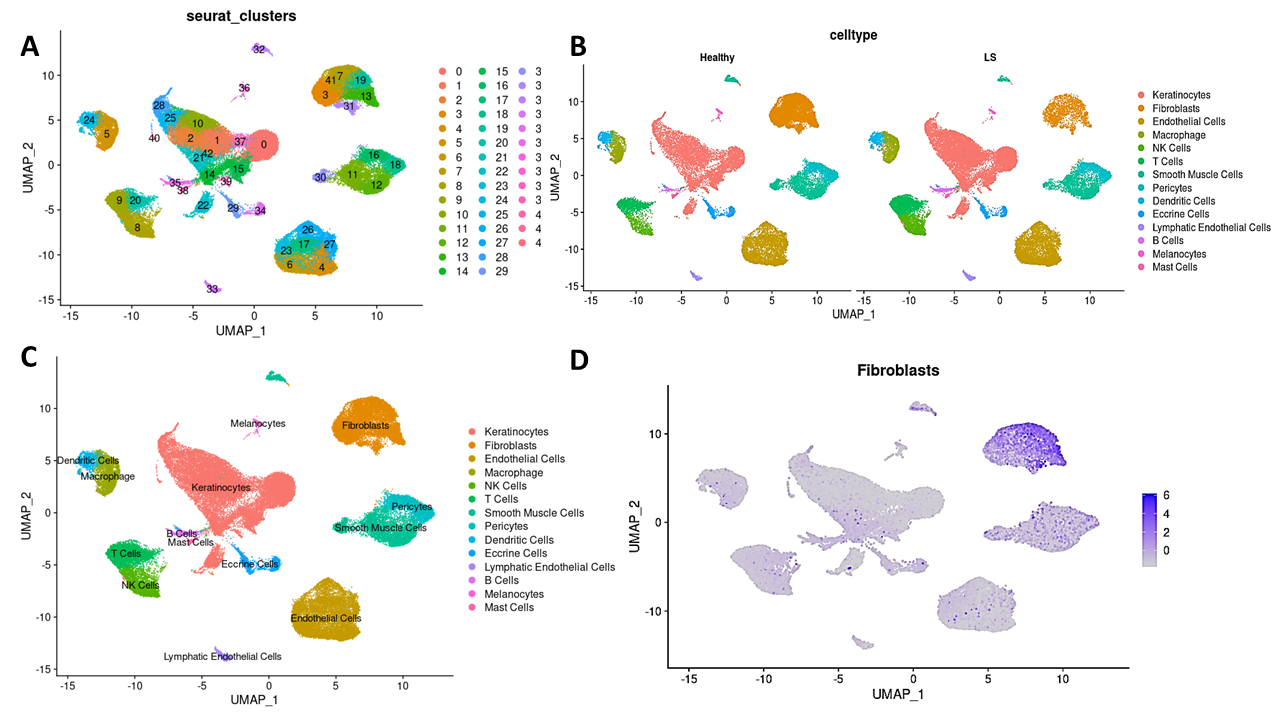

Supplement: Supplementary file 1 [file ijms-24-09796-s001.zip › Figure S1.TIF]

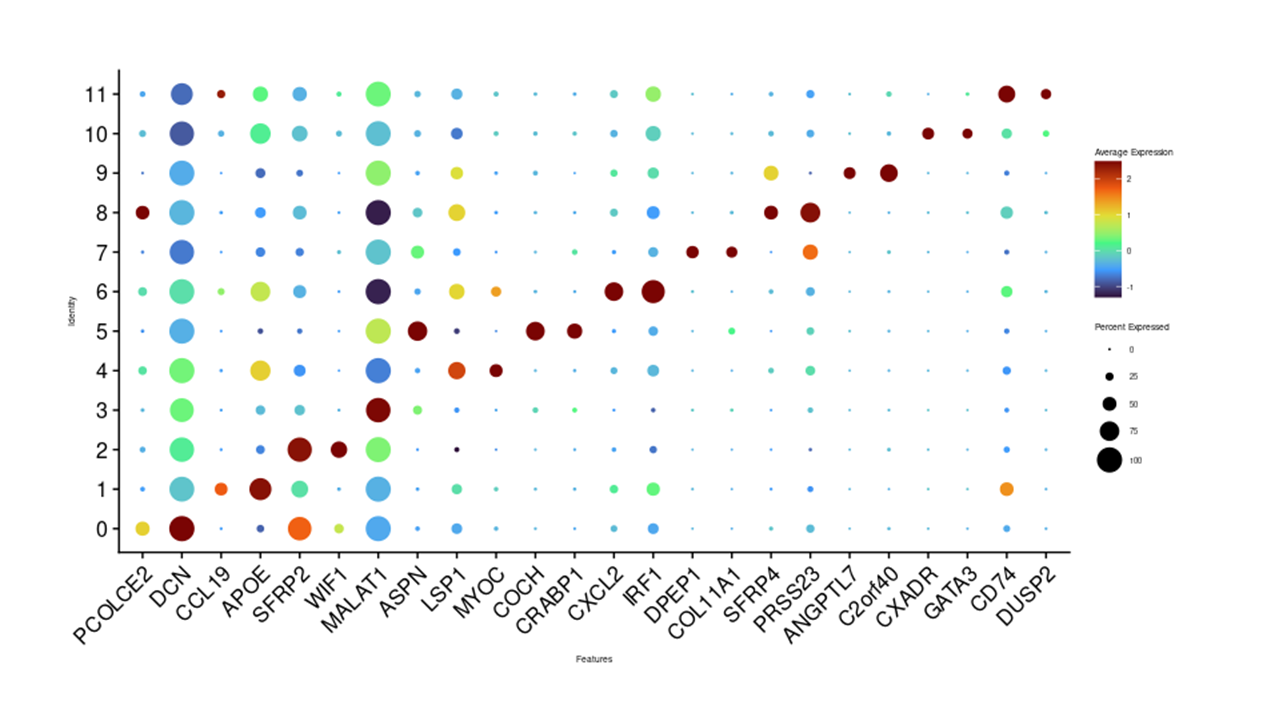

Supplement: Supplementary file 1 [file ijms-24-09796-s001.zip › Figure S2.TIF]

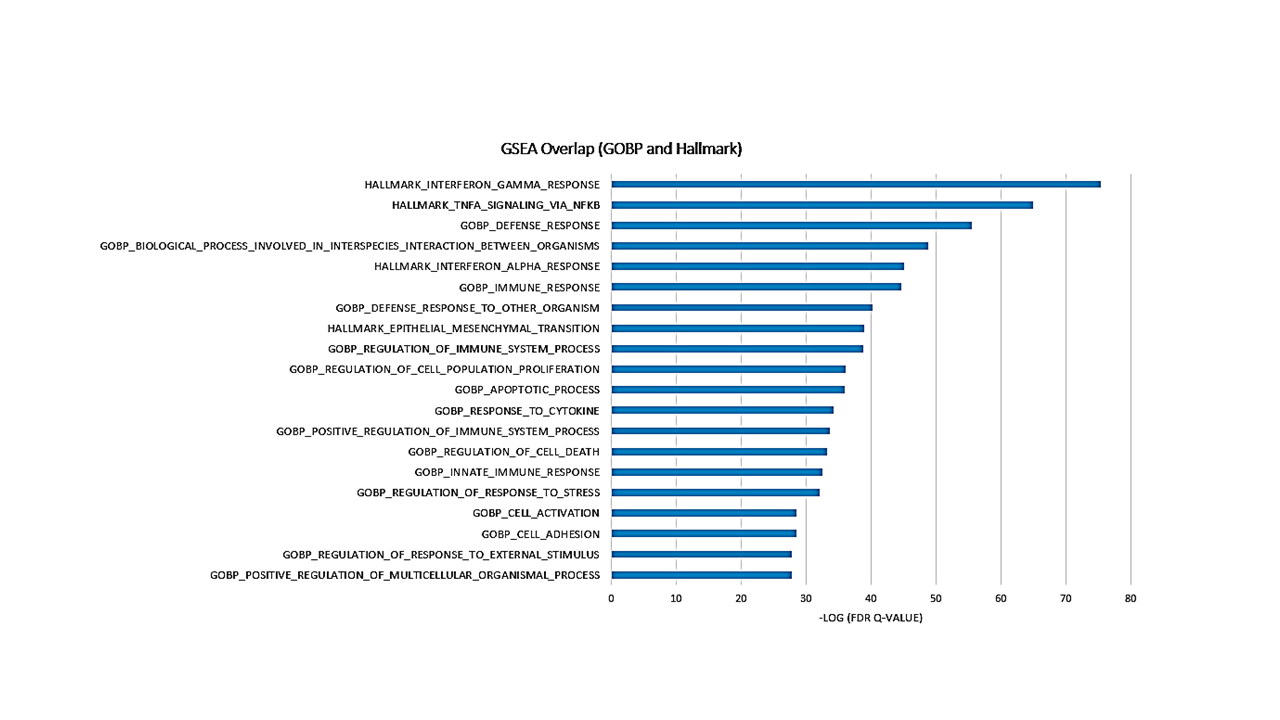

Supplement: Supplementary file 1 [file ijms-24-09796-s001.zip › Figure S3.TIF]

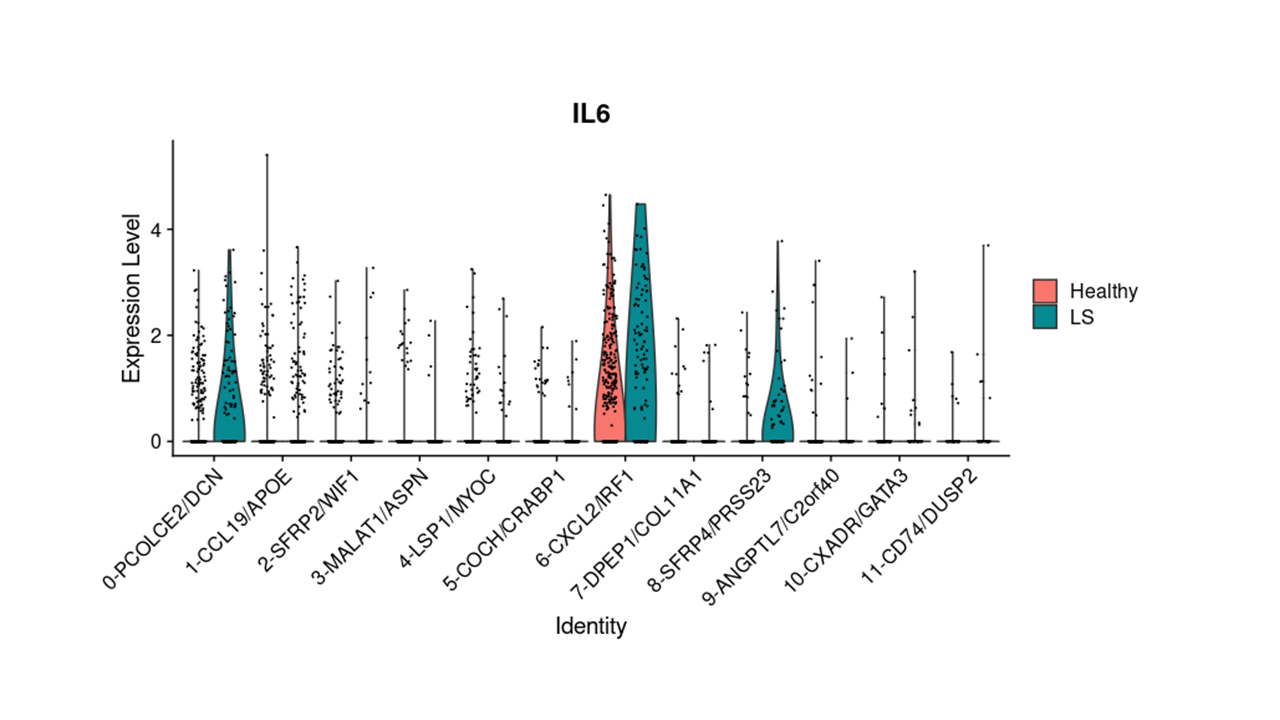

Supplement: Supplementary file 1 [file ijms-24-09796-s001.zip › Figure S4.TIF]

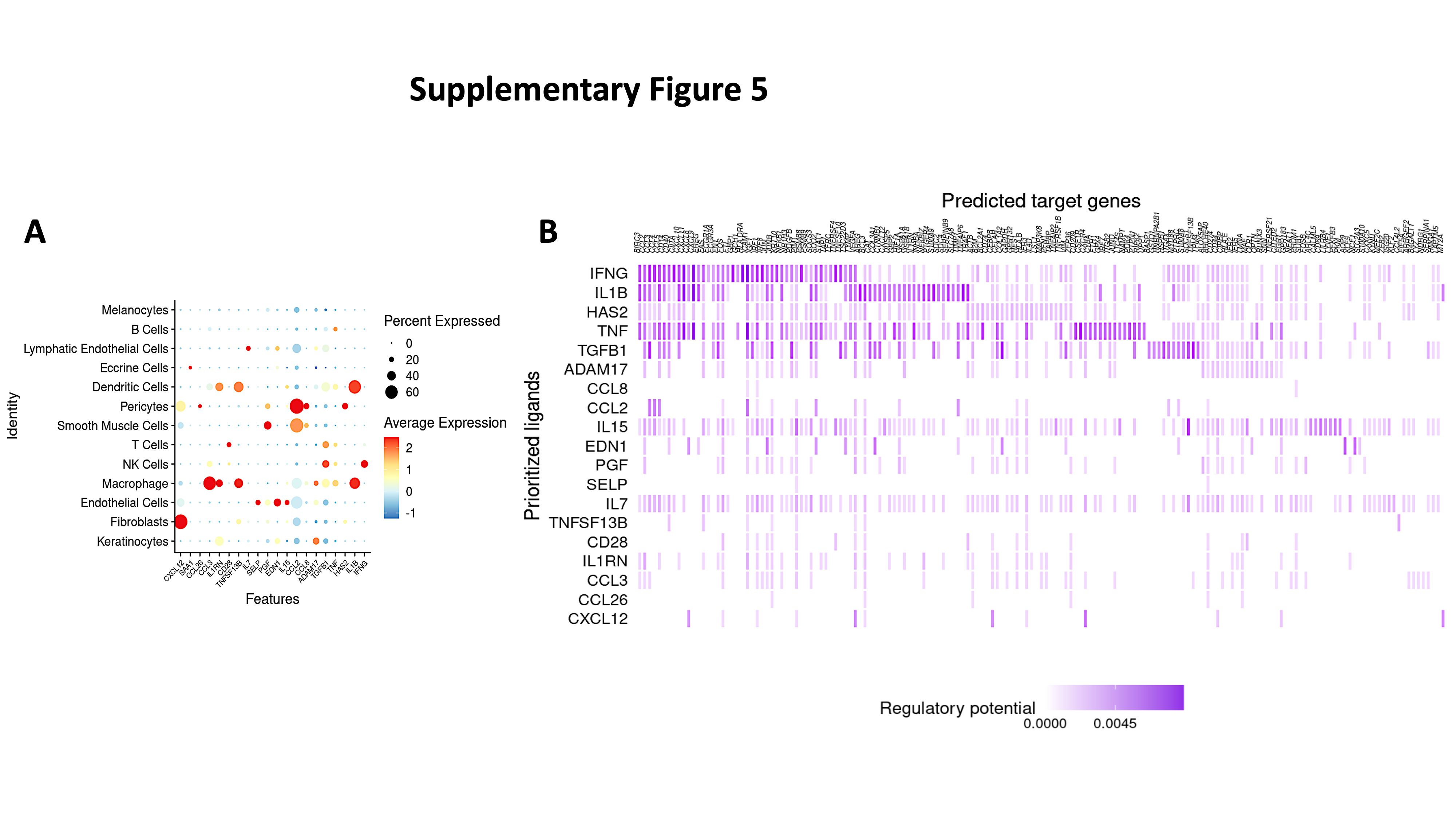

Supplement: Supplementary file 1 [file ijms-24-09796-s001.zip › Figure S5.tiff]
